# Ventricular Volume Load Reveals the Mechanoelastic Impact of Communicating Hydrocephalus on Dynamic Cerebral Autoregulation

**DOI:** 10.1371/journal.pone.0158506

**Published:** 2016-07-14

**Authors:** Christina Haubrich, Marek Czosnyka, Rolf Diehl, Peter Smielewski, Zofia Czosnyka

**Affiliations:** 1 Department of Academic Neurosurgery, Addenbrooke’s Hospital, Cambridge, United Kingdom; 2 Department of Neurology, University Hospital Aachen, Aachen, Germany; 3 Department of Neurology, Alfried-Krupp-Krankenhaus, Essen, Germany; University of Regensburg, GERMANY

## Abstract

Several studies have shown that the progression of communicating hydrocephalus is associated with diminished cerebral perfusion and microangiopathy. If communicating hydrocephalus similarly alters the cerebrospinal fluid circulation and cerebral blood flow, both may be related to intracranial mechanoelastic properties as, for instance, the volume pressure compliance. Twenty-three shunted patients with communicating hydrocephalus underwent intraventricular constant-flow infusion with Hartmann’s solution. The monitoring included transcranial Doppler (TCD) flow velocities (FV) in the middle (MCA) and posterior cerebral arteries (PCA), intracranial pressure (ICP), and systemic arterial blood pressure (ABP). The analysis covered cerebral perfusion pressure (CPP), the index of pressure-volume compensatory reserve (RAP), and phase shift angles between Mayer waves (3 to 9 cpm) in ABP and MCA-FV or PCA-FV. Due to intraventricular infusion, the pressure-volume reserve was exhausted (RAP) 0.84+/-0.1 and ICP was increased from baseline 11.5+/-5.6 to plateau levels of 20.7+/-6.4 mmHg. The ratio dRAP/dICP distinguished patients with large 0.1+/-0.01, medium 0.05+/-0.02, and small 0.02+/-0.01 intracranial volume compliances. Both M wave phase shift angles (r = 0.64; p<0.01) and CPP (r = 0.36; p<0.05) displayed a gradual decline with decreasing dRAP/dICP gradients. This study showed that in communicating hydrocephalus, CPP and dynamic cerebral autoregulation in particular, depend on the volume-pressure compliance. The results suggested that the alteration of mechanoelastic characteristics contributes to a reduced cerebral perfusion and a loss of autonomy of cerebral blood flow regulation. Results warrant a prospective TCD follow-up to verify whether the alteration of dynamic cerebral autoregulation may indicate a progression of communicating hydrocephalus.

## Introduction

In communicating hydrocephalus, the disorders of cerebrospinal fluid- and cerebrovascular circulation can be found associated to one another [1]. The causal link between both is still not known. Pena and colleagues have proposed that the chronic ventricular dilatation in communicating hydrocephalus results from a reversal of interstitial fluid flow into the parenchyma and a reduced tissue elasticity [2]. This may lead to a compression of local vessels and impaired cerebrovascular regulation. A H_2_^15^O PET study by Momijan et al. for instance, has suggested that this interaction may be furthered by the intraventricular volume load [1]. This was suggested by the fact that effects where pronounced in the periventricular parenchyma where compressive effects are stronger than in the juxtacortical areas. Further hints for a direct interaction came from studies comparing clinical findings before and after shunting which revealed that in patients with normal pressure hydrocephalus cerebral blood flow improves after drainage of cerebrospinal fluid (CSF) [3]. Before shunting, a cerebral blood flow increase in response to the CSF withdrawal seemed to be predictive for a clinical improvement after shunting [4,5]. On the contrary, one could assume that the ventricular volume load would deter cerebral perfusion and cerebral blood flow regulation. In order to test this hypothesis we have studied the relationship between peripheral arterial blood pressure and cerebral blood flow velocity before and during controlled ventricular volume load.

The ventricular constant-flow infusion with Hartmann’s solution is a diagnostic means for the evaluation of the dynamics of cerebrospinal fluid circulation. In patients with ventricular dilation of various origin, it has been shown that flow velocity responses to slow spontaneous blood pressure changes can still be detected during ventricular volume load [6,7]. It is applied both, to confirm the diagnosis of normal pressure hydrocephalus as well as to test the dynamics of CSF circulation after shunt insertion [6,8,9]. Volume is infiltrated via the cerebral ventricles or lumbar subarachnoid space, until the ventricular volume reserve is exhausted and the intraventricular pressure reaches a plateau level [9]. This pressure plateau, marks the equilibrium between volume infiltration and CSF resorption [6]. These standards also provide comparable information about the intracranial pressure compliance in patients with communicating hydrocephalus which according to the Monro-Kellie doctrine ultimately is determined by the pressure compliance of all intracranial compartments [10–12]. We hypothesized that if communicating hydrocephalus similarly alters the circulation of CSF and cerebral blood, the degree of alteration may depend on intracranial mechanoelastic properties as the ventricular pressure compliance.

## Methods

**Fig 1:**
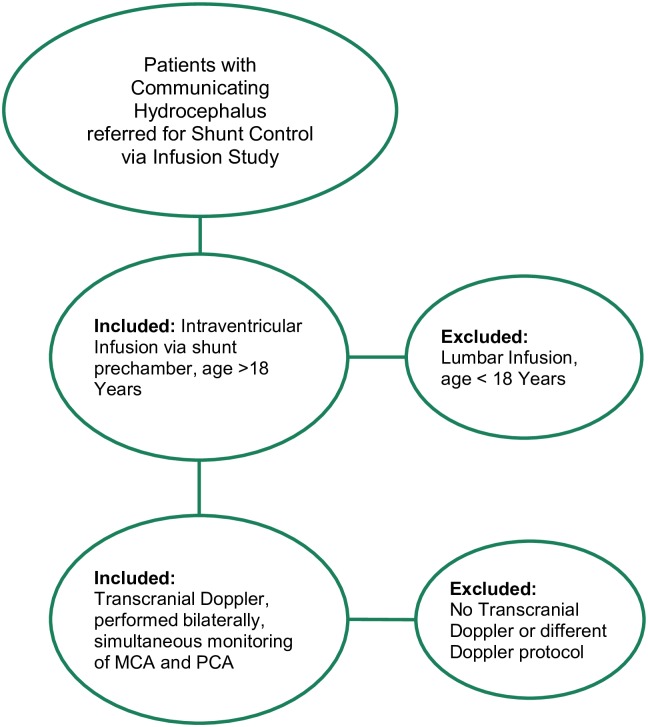
Flow Chart showing inclusion and exclusion criteria in patients with communicating hydrocephalus referred for shunt control via infusion study.

Twenty three patients (12 female, 11 male, mean age 51±17) with shunted hydrocephalus of communicating idiopathic, posttraumatic, and other origin were examined with the CSF infusion test which is routinely performed to obtain measure of the CSF dynamics [9]. The transcranial Doppler (TCD) monitoring in this study was performed in addition to the clinical routine diagnostics in patients with hydrocephalus. It was approved by the National Research Ethics Services (NRES), Committee East of England, Norfolk (ref number LREC 02/308). Written informed individual consent for participation in the study and the scientific use of the data was obtained from patients. Included were patients age >18 years, examined with intraventricular infusion via shunt and simultaneous TCD in the left middle cerebral artery (MCA) and right posterior cerebral artery (PCA). Excluded were patients not examined with TCD or a different TCD protocol (Fig 1).

The infusion studies were performed as a part of clinical routine diagnostics of patients referred to the Hydrocephalus Clinic, Addenbrooke’s Hospital, Cambridge UK for shunt control. The infusion studies are clinical routine in our hospital for 20 years and are performed in more than 300 patients per year. This standard diagnostic procedure in patients with CSF disorders is approved by National Institute of Clinical Excellence, UK (https://www.nice.org.uk/guidance/ipg263). The infusion study data with TCD monitoring was analyzed anonymously as a part of the standard clinical audit. The clinical audit was commissioned by the head of Hydrocephalus Clinic, Addernbrookes Hospital and approved by audit facilitator. In this study patient data were collected during routine care and were analyzed retrospectively.

After 5 minutes of baseline measurements, the ventricular infusion of Hartmann’s solution (standard compound sodium lactate) was initiated at a constant rate of 1.5 ml/min and was continued until a steady-state intracranial pressure plateau (equilibrium between infused and resorbed CSF fluid) was achieved. The infusion was stopped when the intracranial pressure (ICP) reached the plateau level or exceeded 40 mmHg.

The monitoring began at ICP baseline, included the plateau pressure, and was continued after the cessation of infusion, until ICP decreased to steady baseline levels. The monitoring included ICP, arterial blood pressure (ABP), and the cerebral blood flow velocity (FV): The ICP was continuously monitored via a pressure transducer at a Hartmann’s solution-filled tube connected to the intraventricular catheter or shunt antechamber.

The arterial blood pressure was monitored non-invasively using a servo-controlled finger plethysmograph (Finapres 2300, Ohmeda). The hand was kept steady at the level of the heart during the entire recording. The cerebral blood flow velocity (FV) in the left MCA and right PCA were measured with TCD (DWL-MultiDop, DWL). The 2 MHz probes were held in position using a headband (Marc 600, Spencer Technologies, Seattle, USA). A personal computer running the in-house software (ICM+; www.medschl.cam.ac.uk/icmplus) captured the time-averaged values of ICP, ABP, FV, and cerebral perfusion pressure (CPP = ICP-ABP). The data were sampled at a rate of 50Hz and were digitalized. The artefacts were manually removed after recording.

**Fig 2:**
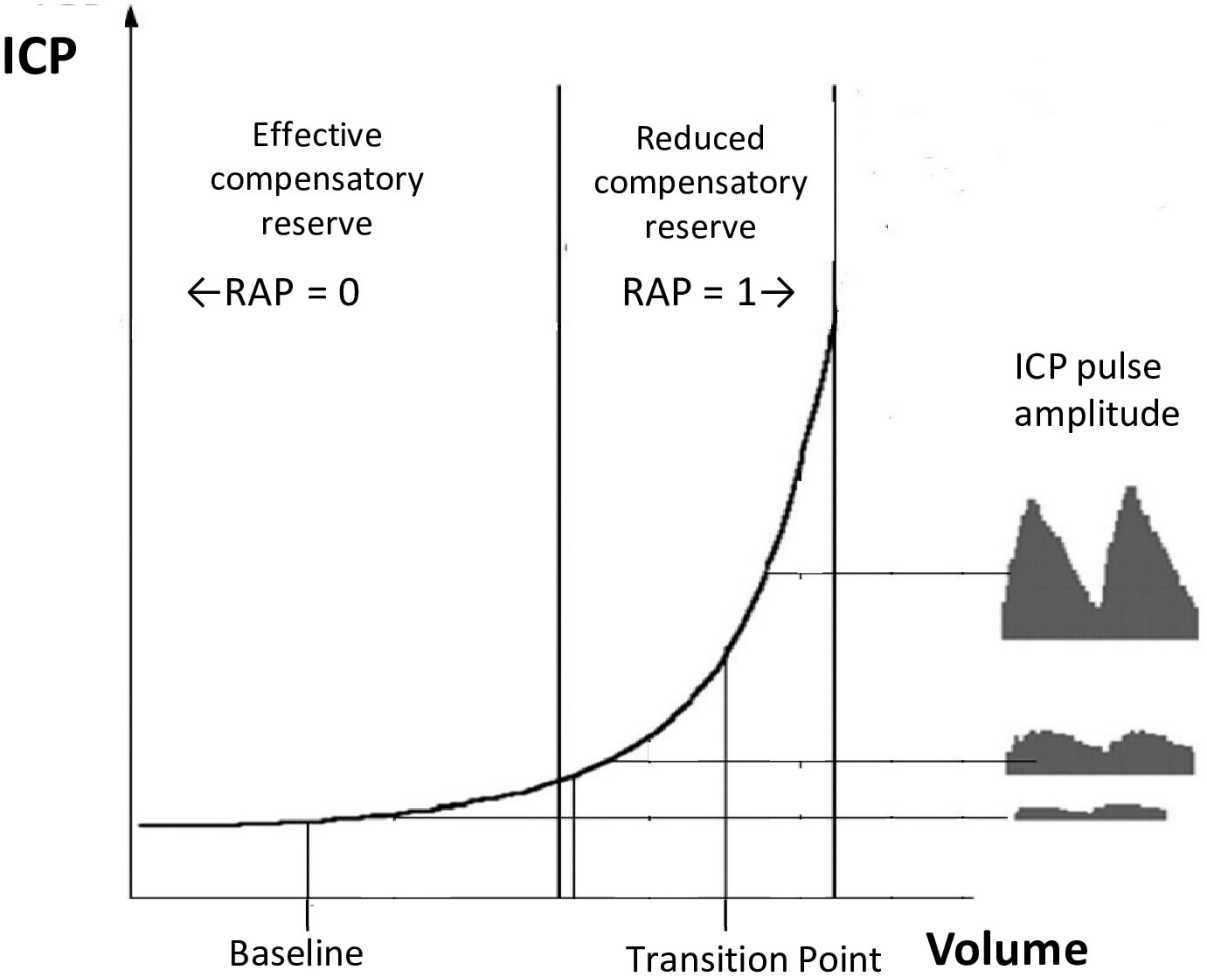
Model of the non-linear relationship between intracranial pressure (ICP) intracranial volume, and the RAP (index of compensatory reserve). The pulse amplitude of ICP is expressed along the y-axis on the right side of the panel and results from pulsatile changes in cerebral blood volume (expressed along the x axis). In the flat zone of the curve compensatory reserve is effective whereas the exponential zone depicts the exhausted compensatory reserve. The transition point marks the upper limit of reserves in the craniospinal system. In the flat zone, the pulse amplitude of ICP is low and does not depend on mean ICP. In the exponential part of the curve, the pulse amplitude increases with the mean ICP due to exhausted compensatory reserve.

The volume-pressure compensatory reserve was assessed using the index RAP [13]. The RAP index is the correlation coefficient (R) between the amplitude (A) of the ICP pulse waves (40–150 cpm) and the mean intracranial pressure (P)) [14]. The RAP is derived by linear correlation between 40 consecutive, data points of peak-to-peak amplitudes of ICP and mean ICP, each data point acquired within a 10s wide time window. Windows were overlapping by 2.5 s. RAP values hence were calculated every 300 seconds. The RAP describes the degree of correlation between the pulse amplitude of ICP and mean ICP over short periods of time (∼5 min). Theoretically, the RAP coefficient indicates the relationship between ICP and changes in intracerebral volume—the ‘pressure–volume’ p/v curve (Fig 2) [15]. The RAP close to 0 indicates a good compensatory reserve, RAP >0.6 indicates a significantly diminished compensatory reserve. Exhausted reserves can be identified for instance, when RAP is above 0.8 slow wave amplitudes are directly dependent on mean intracranial pressure levels [16]. The index RAP, has been shown to provide information about cerebrospinal reserve in hydrocephalus as well as traumatic brain injury [16–19].

While RAP and ICP are defining the working point on the p/v curve the gradient dRAP/dICP measures its slope. Cerebrospinal compliance is proportional to the volume which can be stored till compensatory reserves are exhausted [14]. This is reflected in the difference between the RAP indices at plateau minus baseline (dRAP). The compliance of the cerebrospinal space is inversely proportional to the change in intracranial pressure (plateau—baseline, dICP) [20]. During infusion study, the ICP plateau marks the upper limit of the proportional part of the p/v relationship (Fig 2).

The further analysis was focused on spontaneous oscillations in ABP and TCD flow velocity 3 to 9 cpm (M waves) which are induced by peripheral sympathetic regulation and are transferred to cerebral circulation [21]. The spontaneous regulatory capacity is reflected by the positive phase relation between these oscillations in ABP (input function) and cerebral blood FV (output function). Normal references for phase shift angles are for instance 57.5+/-16.3 grad [21]. A significantly reduced M wave phase shift (<30 grad; p<0.05) is considered as indicative for a diminished regulatory capacity [22]. By means of Fast Fourier transformation M waves were be detected in every ABP- and CBFV-data file. The calculation of phase shift angles was enabled via cross-spectrum analysis. Prerequisite for reliable phase shift measures was a sufficient coherence (≥0.4) between M waves in ABP and CBFV.

All values are given as mean +/- standard deviation. Statistical significance was set at p<0.05. For the comparison between baseline and plateau as well as between subgroups of different intracranial volume compliance we applied the ANOVA using SPSS (12.0.1). The relationship between volume compliance and phase shift angles or CPP, was assessed using linear regression analysis.

## Results

Twenty-three data files depicting time courses of ICP, ABP and FV (MCA and PCA) during 5+/-3 min ICP baseline and during 5+/-3 min ICP plateau were included in the analysis. Via ventricular infusion, the ICP was led to a plateau of 20.7+/-6.4 mmHg. RAP marked the exhaustion of volume compensatory reserve 0.84+/-0.1. For the physiological and derived parameters at baseline and plateau pressure levels please see Table 1.

**Table 1:** Physiological parameters at baseline and plateau levels.

	Baseline	Plateau
ICP [mmHg]	11.46±5.55	20.73±6.39 *
RAP	0.39±0.28	0.84±0.10*
ABP [mmHg]	83.61±16.70	90.04±21.69
CPP [mmHg]	74.77±14.69	76.30±18.59
FV-MCA [cm/s]	61.97±17.40	59.29±18.03
FV-PCA [cm/s]	35.25±8.81	33.12±9.19
Phase-MCA [grad]	44.6±43.80	49.9±45.9
Phase-PCA [grad]	47.5±53.25	42.05±52.6

All values as mean +/- SD. Significant differences (p<0.05) between baseline and plateau are marked as “*”.

**Fig 3:**
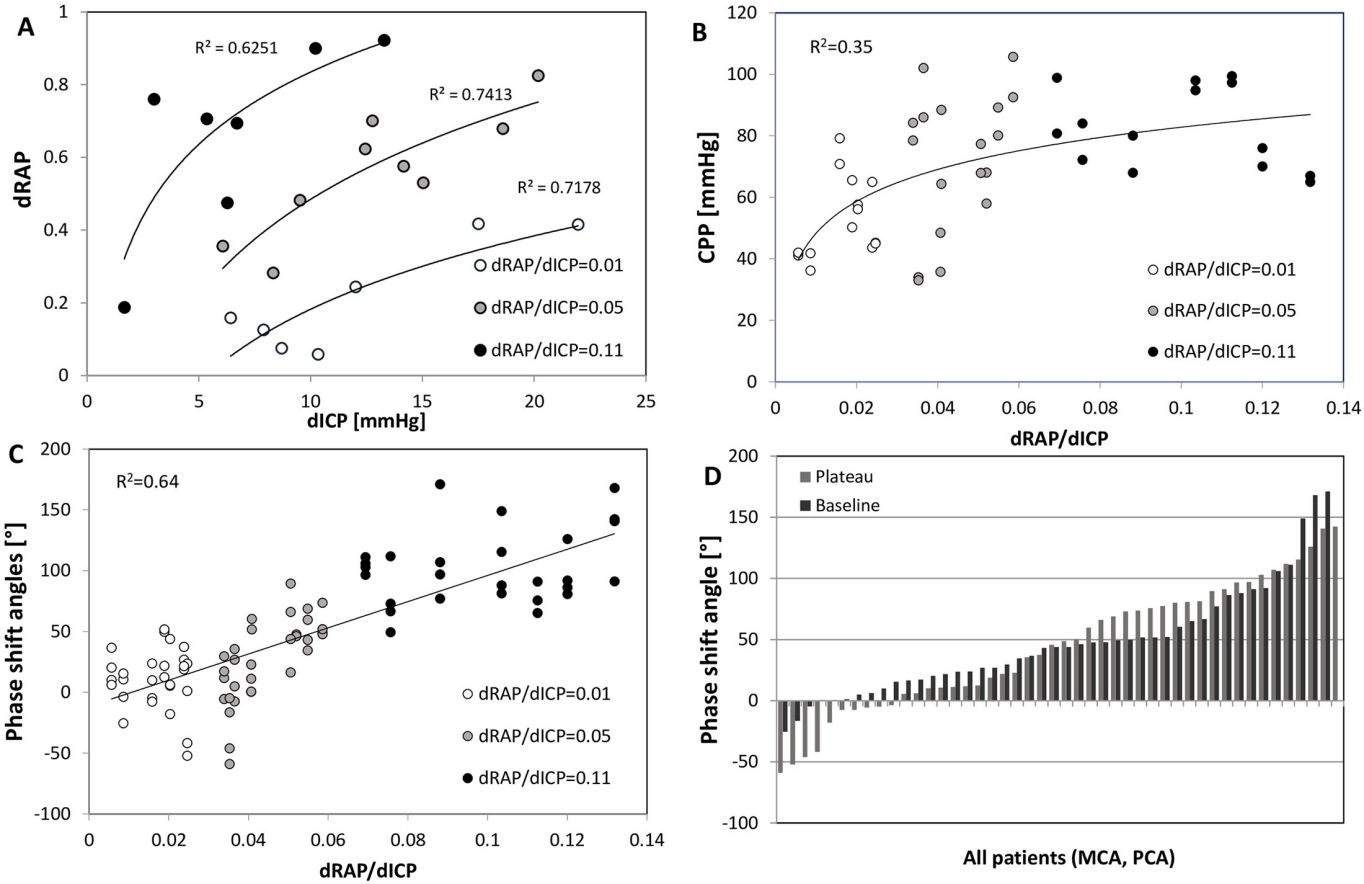
A to D—Relationship between intracranial pressure (ICP), compensatory reserve index (RAP), cerebral perfusion pressure (CPP), and M wave phase shifts. Plotted against each other are dRAP vs dICP, the trend lines mark different slopes, e.g. dRAP/dICP gradients of patients with low (0.01/mmHg), medium (0.05/mmHg), and high (0.11/mmHg) pressure-compliance gradients (A), dRAP/dICP vs. CPP (B), dRAP/dICP vs. M wave phase shift angles (C), and the range of values for phase shift angles across all patients (D).

Unlike RAP and ICP, mean values of ABP, MCA-FV, PCA-FV, CPP as well as MCA- and PCA-phase shift angles did not significantly change between baseline and plateau. Although baseline ICP and RAP varied among patients dRAP and dICP were significantly correlated and suggested similar intracranial compliance among patients of three subgroups (Fig 3A). Responses of RAP and ICP to the ventricular volume load dRAP/dICP indicated large (0.11+/-0.06/mmHg; n = 7), medium (0.05+/-0.01/mmHg in n = 9 and low 0.01+/- 0.01/mmHg, n = 7) intracranial volume-compliances.

**Fig 4:**
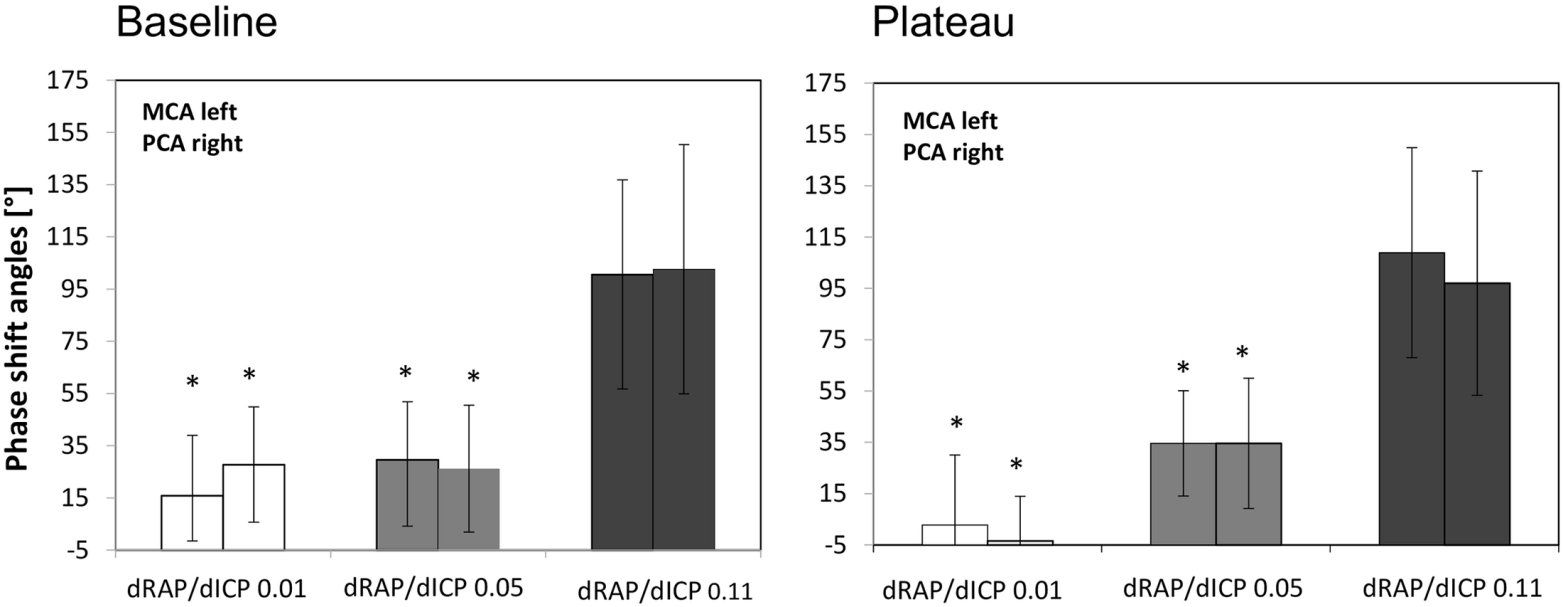
A and B—Comparison between patient subgroups with low, medium, and high pressure compliances at baseline (A) and plateau (B). M wave phase shift values in patients with low and medium pressure compliance e.g. gradients dRAP/dICP of 0.01 and 0.05/mmHg were significantly lower than in patients with gradients dRAP/dICP of 0.11/mmHg. Significant differences were marked with “*”.

Phase-shift angles between M waves in ABP and MCA-FV as well as between ABP and PCA-FV were ranging between -58.9 and +171.0grad (Fig 3D). Lower dRAP/dICP ratios, were associated with gradually lower phase shift angles (phase shifts at baseline as well as plateau pressure levels; r = +0.64; p<0.05 Figs 3C, 4A and 4B) and lower CPP values (CPP at baseline as well as plateau pressure levels; r = +0.35; p<0.05; Fig 3B). There were no significant differences between phase shifts angles calculated for the MCA- and PCA-territories (Fig 4A and 4B).

The comparison of phase shifts between baseline and plateau in most cases revealed no significant differences. Under conditions of low pressure compliance of 0.01+/-0.01/mmHg only, the ventricular volume load led to a decline of M wave phase shift in the PCA.

## Discussion

This study showed that cerebral perfusion pressure and dynamic cerebral autoregulation in patients with communicating hydrocephalus are depending on the ventricular pressure compliance. CPP and M wave phase shift angles at baseline as well as during controlled ventricular volume load were correlated with dRAP/dICP. The transient ventricular volume load did however, neither alter the dynamic cerebral autoregulation nor CPP significantly. There are many hypotheses about the link between communicating hydrocephalus and alterations of cerebral blood flow and perfusion [23–27]. Imaging studies have shown that patients with communicating hydrocephalus may present chronic ischemic lesions in the periventricular white matter [28]. While some have presumed that the parenchymal lesions drive the process of ventricular enlargement in communicating hydrocephalus others have proposed that reduced brain tissue perfusion and blood flow are consequences of ventricular volume overload, increased CSF pulse pressure, periventricular edema, and periventricular shear stress [1,29,30]. The findings of Momjan et al. (2004) suggested that in communicating hydrocephalus, the cerebral perfusion follows an abnormal U-shaped gradient with lowest levels at the lateral ventricles and the outermost subcortical white matter [1]. Their imaging studies with H2O positron emission tomography seemed to mirror the effect of the tissue pressure gradient predicted by finite poroelastic models [2,30]. Momjan et al (2004) observed a particular vulnerability of the cerebral blood flow in the paraventricular watershed region of the corona radiata were poroelastic models of communicating hydrocephalus predicted the maximum tissue pressure due to ventricular volume overload [1].

By means of the TCD-based analysis of dynamic cerebral autoregulation in patients with communicating hydrocephalus we did not find any regional differences between the M wave phase shifts in the anterior and posterior parts of circulation. The advantage of TCD lies in the time resolution of seconds and less. The method thus detects gradual differences of the autoregulatory response to spontaneous blood pressure oscillations of a frequency of 3 to 9 cycles per minute. M wave phase shifts may depend on the degree of carotid or MCA stenosis as well as the sufficiency of collateral blood supply [31–33]. Our results in communicating hydrocephalus, showed a direct link of M wave phase shifts angles with dRAP/dICP. This indicated that a lower cerebrospinal compliance is associated with the loss of cerebrovascular autonomy. Moreover, patients with lowest intracranial pressure compliance also had lowest CPP values. Our study suggested that in communicating hydrocephalus the intracranial compliance is a determining factor for the cerebral blood flow regulation.

Mase et al (2006) hypothesized that indeed, the most important factor in the underlying pathophysiology of communicating hydrocephalus is the alteration of intracranial compliance [34]. We have assessed the cerebrospinal compliance via the controlled intraventricular volume load. The ventricular infusion was always terminated as soon as the upper limit of cerebrospinal reserve was reached which is indicated via the compensatory reserve RAP index close to “+1” [14]. Although the starting volume can shift RAP the volume would not alter the slope of the p/v curve. This is supported by our observation dRAP and dICP were significantly correlated among three patient subgroups.

The gradients dRAP/dICP distinguished subgroups of low, medium, and high pressure compliance in patients with communicating hydrocephalus and provided information about the mechanoelastic characteristics of the cerebrospinal system [13]. Recently, imaging studies with magnetic resonance elastography (MRE) gave visual evidence for the permanent alteration of mechanoelastic characteristics under conditions of communicating hydrocephalus [35]. These studies depicted that the ventricular shear modulus had increased to a level comparable to brain tissue [36]. Like Momjans earlier observations also MRE appears to support the prediction of finite poroelastic models: These models are based on the Monro-Kellie doctrine which takes into account that the compliance of all intracranial compartments is limited by the skull [10–12]. A ventricular volume overload would hence increase the pressure especially in the periventricular brain tissue. Chronic ventricular volume overload and shear stress may not only lead to stiffening of intracranial ventricles but may lead to the direct interaction between ventricles and brain parenchyma. We believe that the loss in autonomy of the cerebrovascular response in communicating hydrocephalus, could be a consequence of a direct compartmental interaction which limits the transfer of spontaneous oscillations from systemic blood pressure to MCA and PCA flow velocity. Hence, the lowest ventricular pressure compliance in our study was associated with an almost pressure passive transfer of spontaneous oscillations to the cerebral vasculature which indicated an impaired autoregulation.

This study showed that cerebral perfusion pressure and dynamic cerebral autoregulation in particular, depend on the cerebrospinal pressure compliance. These results suggested that the alteration of mechanoelastic characteristics contributes to a reduced cerebral perfusion and a loss of autonomy of cerebral blood flow regulation. Results warrant a prospective study with TCD and MRI in order to investigate whether impaired dynamic cerebral autoregulation and low cerebrospinal compliance in communicating hydrocephalus are related to small vessel disease.

## Abbreviations

ABP: Arterial Blood Pressure
CPP: Cerebral Perfusion Pressure
CSF: Cerebro-Spinal Fluid
FV: Flow Velocity
ICP: Intra-Cranial Pressure
MCA: Middle Cerebral Artery
PCA: Posterior Cerebral Artery
p/v-curve: pressure-volume curve
RAP: Index of pressure-volume compensatory reserve
TCD: Trans-Cranial Doppler
